# Enhancing Patient Care II: The Clinical Impact of Medical Information Services

**DOI:** 10.1007/s43441-022-00385-1

**Published:** 2022-03-15

**Authors:** Leena Jindia, Sharone Keane, Susan Wnorowski, Evelyn R. Hermes-DeSantis

**Affiliations:** 1 Medical Information Content Strategy & Innovation, Janssen Scientific Affairs, LLC, Titusville, NJ USA; 2 Worldwide Research and Development, Global Medical Information, Pfizer, Inc., New York, NY USA; 3 US Medical Information, US Medical Affairs Division, Ipsen Biopharmaceuticals, Inc., Basking Ridge, NJ USA; 4 Research and Publications, phactMI, West Point, PA USA; 5 Rutgers The State University of New Jersey, Piscataway, NJ USA

**Keywords:** Clinical impact, Medical information, Survey, Healthcare professional

## Abstract

**Background:**

Understanding the ways that healthcare providers (HCPs) utilize medical information received from the pharmaceutical industry is important so that the information can be tailored and customized to meet the HCPs needs. Additionally, this understanding supports the value of the information provided. The purpose of this study was to collect opinions of HCPs who recently requested information from a manufacturer’s Medical Information (Med Info) Service. HCPs provided their opinions on the perceived quality, relevance, impact on patient care, and intended usage of information.

**Methods:**

HCPs who recently requested medical information from one of eight participating companies received a Survey Monkey link in the information response. Data collected included demographics, perceived quality, relevance, impact on patient care, and intended usage of the information. Data were analyzed via descriptive statistics.

**Results:**

Over a 14-month period, 246 HCPs responded to the survey. Eight companies participated in the survey. Customer responses to the survey ranged from 2 to 97 per company. A total of 99 pharmacists, 68 physicians, 22 registered nurses, 21 nurse practitioners, 8 physician assistants, and 28 others participated in the survey. Most HCPs (208/227, 92%) contacted the company Med Info Group 1–5 times in the last six months and 67% (159/238) had been in practice greater than 10 years. Most HCPs rated the following quality areas as a 4 or 5 on a 5-point Likert scale: timeliness (195/225, 87%), trustworthiness (189/221, 86%), conciseness (185/221, 84%), clarity (180/222, 81%), relevance (178/223, 80%), and completeness (173/222, 78%). The most common reason for contacting Med Info Services was to advance knowledge or education (110/228, 48%). Additional reasons were at the point of care (60/228, 26%), for a specific patient (not at point of care) (60/228, 26%), and to reflect on a treatment decision (59/228, 26%). The relevance of the information provided was utilized for the HCPs own education (99/226, 44%), shared with peers (91/226, 40%), or used for future treatment decisions (88/226, 39%). The information provided enhanced patient care by enabling the HCPs to educate patients more effectively (86/222, 39%), efficacy of treatment regimen was enhanced (70/222, 32%), or other positive impact (65/222, 29%).

**Conclusion:**

The opinions of HCPs who are using Medical Information Services are overall positive. All the quality indicators were rated as a 4 or 5 by the majority of HCPs, with the lowest in completeness (173/222, 78%) and the highest in timeliness (195/225, 87%). Medical Information Services were utilized to advance knowledge/education of the HCP, followed closely by the care of a current or future patient. However, when queried on the relevant use of the information in their practice, the most common answers were for their own education or to share with peers. The impact on patient care was focused on enabling the HCP to educate patients more effectively. The value of medical information is difficult to quantify. Understanding the quality assessment, utilization, and the impact on patient care by HCPs can provide a broad descriptor of value. This study supports the value of the medical information responses provided by pharmaceutical companies to HCPs in their practice(s).

**Supplementary Information:**

The online version contains supplementary material available at 10.1007/s43441-022-00385-1.

## Introduction

Healthcare professionals (HCPs) access and utilize information concerning medications to make informed treatment decisions to better care for their patients. One way for HCPs to find or further clarify information concerning medications is to contact pharmaceutical companies directly [1]. The Medical Information (Med Info) Groups within pharmaceutical companies provide fair and scientifically balanced, current, and accurate medical information responses to unsolicited requests from HCPs, in a timely fashion [2]. These Med Info Groups maintain metrics that may include response time from intake to fulfilment, number of medical information requests, and the types of requests received [3]. Many Med Info Groups also conduct routine surveys to validate if the HCP was satisfied with the response and the timeliness of the response [4].

phactMI is a collaboration of pharmaceutical company Medical Information leaders that are dedicated to supporting HCPs in their commitment to provide quality patient care (phactMI.org). This group has previously gained insights into identifying the sources where HCPs go to find medical information [6]. In a survey conducted by Fung et al. 192 HCPs indicated that the medical information received from four pharmaceutical companies impacted treatment management, prescribing decisions, discussions with patients, future treatment decisions/discussions, or was used for self-education [5]. In another phactMI survey, HCPs stated that the information they received from pharmaceutical companies Med Info Groups were of high quality, useful, and the information provided a clinical impact or a patient benefit [7]. However, that survey was generic in that it was asking HCPs to recall the information they had received at some point in the last year along with their utilization of the information. It is important for the Med Info Groups to know how the information is being used and if there is any impact on clinical outcomes, so they can better tailor responses to align with the HCP’s purpose(s) for seeking this information.

In this follow-up study, the objective was to collect survey data regarding HCP’s opinions on the perceived quality, relevance, impact on patient care, and intended usage of responses they received to their current inquiry to a specific Medical Information Service.

## Methods

A working group from phactMI consisting of Medical Information leaders from eight member pharmaceutical companies created a nine question Internet-based survey. Health care professionals (HCPs) who requested medical information received a company specific survey link in the response document to their inquiry. The survey was open for 14 months. No identifying information was collected during the survey. A copy of the survey is included in Appendix A. This survey was considered a quality improvement project and therefore no IRB approval was necessary.

It was at the discretion of each pharmaceutical company as to which responses and channels included the link. When the link was included, it was for all responses for the specific question for the given channel. The pharmaceutical companies participating in the survey were a representative sample across the spectrum of large to small companies.

HCPs, including physicians, pharmacists, nurse practitioners, registered nurses, physician assistants, and others received the survey when requesting medical information from participating companies.

In addition to demographics, information collected in the survey included assessment of quality factors, and the perceived value, clinical impact, and intended use of the information. The survey consisted of nine questions and would take up to ten minutes to complete. The questions included multiple choice, select all that apply, and 5-point Likert scales. The survey questions were similar to those reported in Albano et al. [7]; however, there were no free text options to specify additional information. Survey data were analyzed using descriptive statistics, including Mann Whitney U tests for ordinal data and Chi Square for nominal data. Any differences and trends amongst HCP types were assessed to highlight areas and opportunities for improvement of Medical Information Services.

## Results

### Demographics

**Fig. 1:**
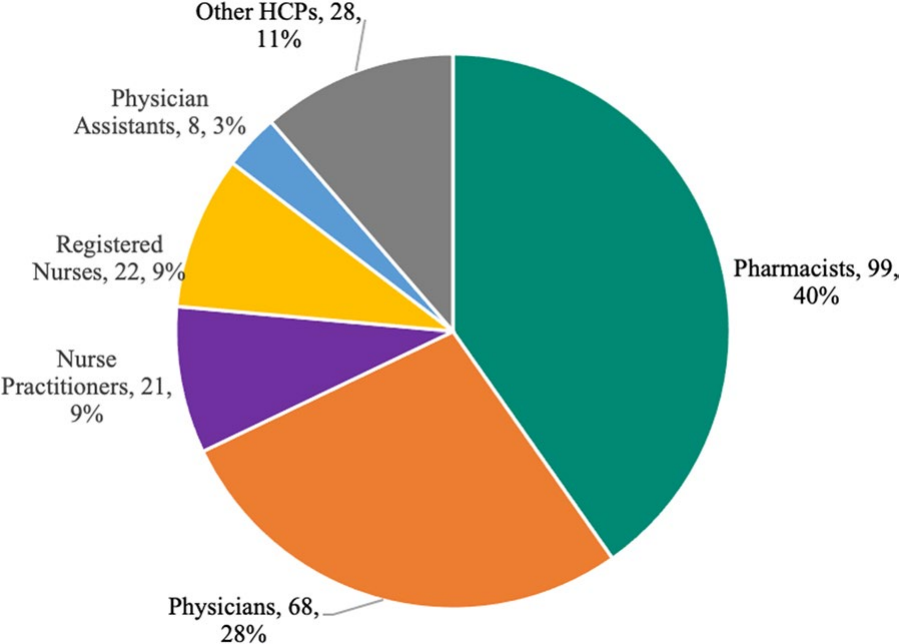
Breakdown of HCP by type

From July 2019 to September 2020, eight participating pharmaceutical companies distributed the survey links. A total of 246 HCPs responded to the survey including 99 pharmacists, 68 physicians, 22 registered nurses, 21 nurse practitioners, 8 physician assistants, and 28 other HCPs (Fig. 1). Eight companies participated in the survey, representing the spectrum of large to small companies. Customer responses to the survey ranged from 2 to 97 per company. As the total number of response documents distributed is unknown, the true response rate is unclear; however, the distribution of HCP types and responses are in line with expectations from Medical Information Services. In terms of practice settings, it was evenly distributed among private practice (53/246, 22%), academic/teaching hospital (48/246, 20%), outpatient clinical practice (48/246, 20%), hospital-pharmacy (39/246, 16%), and community hospital (35/246, 14%). A majority, 67% (159/238), had been in practice greater than 10 years. Demographics are presented in Table 1. In addition, most participants (208/227, 92%) identified as only having contacted Medical Information Service between one and five times in the last six months. For each of the following sections, there were no differences seen among the eight companies.

**Table 1:** Healthcare Providers (HCPs) Baseline Characteristic

Specialty practice	HCPs N = 246	Pharmacists N = 99 40%	Physicians N = 68 28%	NP N = 21 9%	RN N = 22 9%	PA N = 8 3%	Other HCPs N = 28 11%
Primary care	37	12	15	6	1	2	1
Cardiology	14	2	7	2	2	1	0
Oncology/hematology	40	26	6	2	3	0	3
Psychiatry	24	5	8	4	4	1	2
Other	129	53	32	7	12	4	21
*Practice setting (select all)*							
Private practice	53	2	34	9	1	4	3
Academic/teaching hospital	48	19	17	3	7	1	1
Community hospital	35	13	10	5	3	0	4
Outpatient clinic	48	15	9	8	8	3	5
Hospital pharmacy	39	37	–	–	–	–	2
Other	67	31	8	5	9	1	13
*Years in practice*	N = 238	N = 97	N = 66	N = 21	N = 21	N = 8	N = 25
11 year or more	159	51	57	11	18	6	16
10 years or less	79	46	9	10	3	2	9

### Quality Factors of Responses

**Fig. 2:**
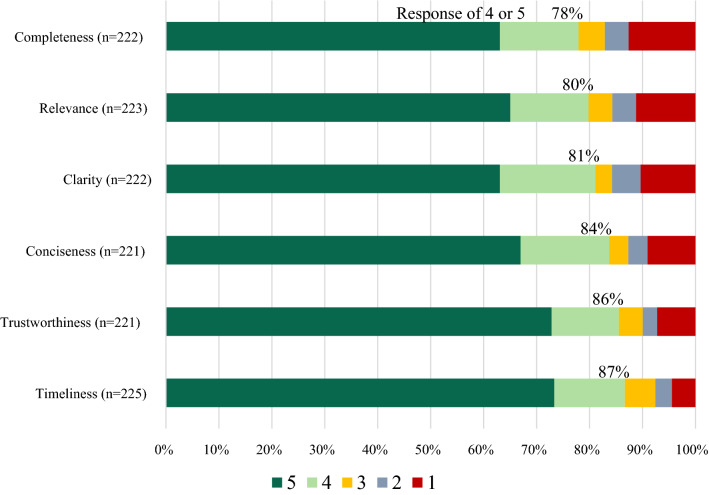
Quality rating of medical information responses—all companies

Participants were asked to rate six factors of quality on a 5-point Likert scale, with 1 being lowest quality and 5 being highest quality. A majority rated all the quality indicators as a 4 or 5: timeliness (195/225, 87%), trustworthiness (189/221, 86%), conciseness (185/221, 84%), clarity (180/222, 81%), relevance (178/223, 80%), and completeness (173/222, 78%). (See Fig. 2) There were no significant differences utilizing the Mann–Whitney *U* test between HCP types or years in practice and the rating of the various quality indicators. Using the Mann–Whitney *U* test, there were also no significant difference among the eight companies and the quality indicators.

### Reason for Contacting Medical Information Services

**Fig. 3:**
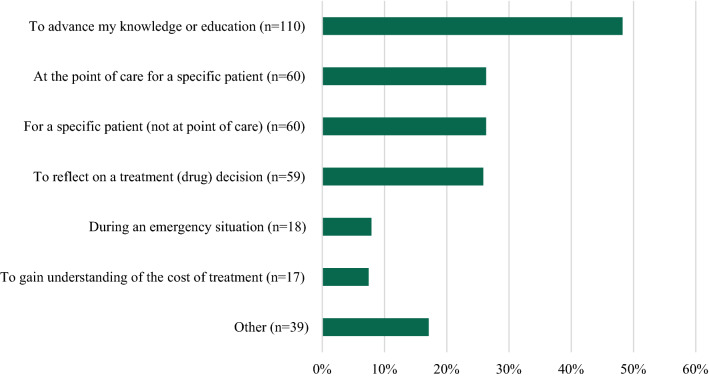
Reasons for requesting information—all companies (multiple responses accepted, N = 228)

The most common reason for contacting Med Info Services was to advance the HCPs’ knowledge or education (110/228, 48%). HCPs were allowed to select multiple responses for this question. Additional reasons were at the patient point of care (60/228, 26%), for a specific patient (not at point of care) (60/228, 26%), to reflect on a treatment decision (59/228, 26%), during an emergency situation (18/228, 8%), to gain understanding of the cost of treatment (17/228, 7%), or other (39/228, 17%). (See Fig. 3) While there were numerical differences between HCP types, there were no statistically significant differences identified via the Chi Square test.

### Relevance of Information from Medical Information Services

**Fig. 4:**
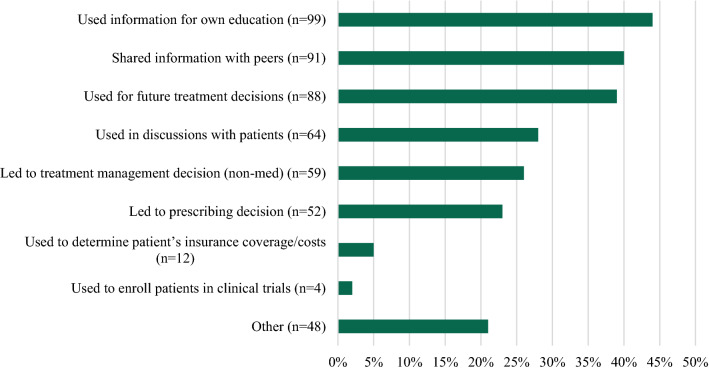
Relevance of information to practice—all companies (multiple selections allowed) (N = 226)

The relevance of the information provided was utilized for the HCPs’ own education (99/226, 44%), shared with peers (91/226, 40%), used for future treatment decisions (88/226, 39%), used in discussion with patients (64/226, 28%), led to a treatment management decision other than prescribing (59/226, 26%), led to a prescribing decision (52/226, 23%), other (48/226, 21%), used to determine patient’s insurance coverage or out-of-pocket costs (12/226, 5%), and used to enroll patients in a clinical trial (4/226, 2%). (see Fig. 4) There were a number of statistically significant difference seen between HCP type and the relevance of the information for their practice (see Table 2). A trend was noted with nurse practitioners reporting a higher relevance of the information leading to a prescribing decision and used in discussions with a patient compared to pharmacists and physicians.

**Table 2:** Relevance of information by HCP type [*N*(%)]

	All HCPs N = 226	Pharmacists N = 88	Physician N = 64	NP/APN N = 21	RN N = 22	PA N = 6	Other HCP N = 25
Used information for own education	99 (44)	39 (44)	25 (39)	13 (62)	10 (45)	2 (33)	10 (40)
Shared information with peers	91 (40)	49 (56)	13 (20)^†‡^	10 (48)	11 (50)	1 (17)	7 (28)^€^
Used for future treatment decisions	88 (39)	34 (39)	27 (42)	12 (57)	6 (27)^†^	2 (33)	7 (28)^†^
Used in discussion with patients	64 (28)	18 (20)*	16 (25)^	13 (62)	8 (36)	2 (33)	7 (28)^†^
Led to treatment management decision	59 (26)	27 (31)	14 (22)*	11 (52)	3 (14)*	0	4 (16)*
Led to prescribing decision	52 (23)	11(13)*	16 (25)*	14 (67)	4 (18)*	3 (50)	4 (16)*
Used to determine patient’s insurance coverage/out of pocked costs	12 (5)	5 (5)	1 (2)	2 (10)	2 (9)	1 (17)	1 (4)
Used to enroll patients in clinical trials	4 (2)	2 (2)	1 (2)	1 (5)	0	0	0
Other	48 (21)	19 (22)	10 (16)	3 (14)	7 (32)	0	9 (36)

*p < 0.001 vs. NP

^*p* < 0.002 vs. NP

^†^*p* < 0.05 vs. NP

^‡^*p* < 0.0001 vs. Pharmacists

^€^*p* < 0.05 vs. Pharmacists

### Impact of Information on Patient Care

**Fig. 5:**
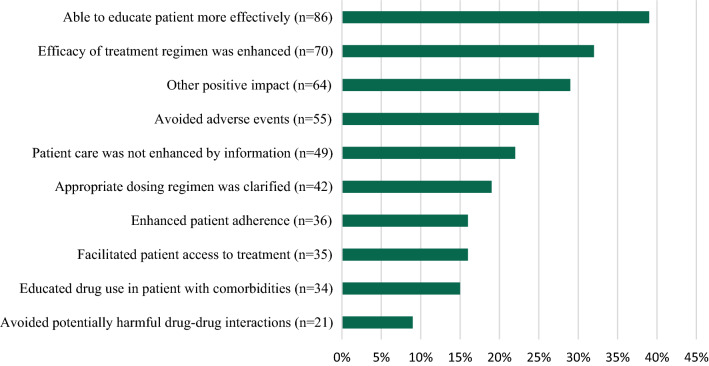
Enhancement/impact on patient care—all companies (multiple selections allow, N = 222)

The HCP’s ability to educate patients more effectively (86/222, 39%) was identified as the most common impact to patient care. Additionally, HCPs indicated that the information enhanced treatment efficacy (70/222, 32%), had another positive impact (65/222, 29%), allowed for the avoidance of an adverse event (55/222, 25%), clarified appropriate dosing regimen (42/222, 19%), enhanced patient adherence (36/222, 16%), facilitated access to treatment (35/222, 16%), provided education concerning use in patients with comorbidities (34/222, 15%), and avoided potentially harmful drug-drug interactions (21/222, 9%). (See Fig. 5) A total of 22% (49/222) indicated that patient care was not enhanced by the information; however, nine of those also indicated positive effects (multiple answers selected: 5 efficacy enhanced, 4 avoided adverse events, 4 avoided drug-drug interactions, 6 educated patients more effectively, 3 clarified dosing regimens, 4 educated about drug use in comorbidities, 3 facilitated treatment access, 5 enhanced adherence, or 6 other positive impact). The reason for requesting the information in the remaining 40 responses of no positive impact were for the HCP’s own education (13), emergency setting (4), at the point of care (9), not at the point of care (10), to reflect on treatment decision (11), and cost (1). Additionally, the relevance of the information in these cases were identified as led to treatment management decision (8), led to prescribing decision (6), used for future treatment decision (11), used in discussion with patient (11), used for own education (18), and shared information with peers (11).

There were several statistically significant differences seen between HCP type and the impact of the information (see Table 3). The trend of differences seen between nurse practitioners and physicians and pharmacists were in enhancing efficacy, clarifying dosing regimens, and educating patients.

**Table 3:** Enhancement of patient care (N (%))

	All HCPs N = 222	Pharmacists N = 85	Physician N = 64	NP/APN N = 21	RN N = 21	PA N = 6	Other HCP N = 25
Efficacy of treatment regimen enhanced	70 (32)	21 (25)^*^	19 (30)^^^	16 (76)	6 (29)^†^	3 (50)	5 (20)^^^
Avoided adverse events	55 (25)	20 (24)	14 (22)	9 (43)	4 (19)	1 (17)	7 (28)
Avoided potentially harmful drug-drug interactions	21 (9)	6 (7)^‡^	5 (8) ^‡^	5 (24)	1 (5)	2 (33)	2 (8)
Able to education patients more effectively	86 (39)	25 (29)^†^	25 (39)^‡^	14 (67)	11 (52)^#^	3 (50)	8 (32)^‡^
Appropriate dosing regimen clarified	42 (19)	15 (18)^†^	7 (11)^^^	10 (48)	5 (24)	2 (33)	3 (12)^†^
Educated about drug use in patients with comorbidities	34 (15)	8 (9)^†^	11 (17)	6 (29)	4 (19)	1 (17)	4 (16)
Facilitated patient access to treatment	35 (16)	10 (12)	9 (14)	6 (29)	6 (29)	2 (33)	2 (8)
Enhanced patient adherence	36 (16)	13 (15) ^#^	6 (9)	5 (24)	5 (24)	0	7 (28)
Other positive impact	65 (29)	32 (38)	15 (23)^#^	4 (19)	2 (10)	0	12 (48)
Patient care was not enhanced	49 (22)	21 (25)	13 (20)	4 (19)	7 (33)	0	4 (16)

^*^p < 0.00001 compared to NP

^*p* < 0.001 compared to NP

^†^*p* < 0.01 compared to NP

^‡^*p* < 0.05 compared to NP

^#^*p* < 0.05 compared to other HCP

## Discussion

The opinions of HCPs who are using Medical Information Services were positive in terms of quality of response and the impact the information had on patient care. Overall, this research confirms the results of Albano et al. [7] in a different setting. Albano’s research was of a general HCP population asked for their opinions on information received over the last 12 months from a pharmaceutical company. This research utilized similar survey questions; however, the HCPs included received the survey link with the response to an inquiry they had placed with specific pharmaceutical Medical Information Services. The major differences in the two surveys are (1) real-time surveying in proximity to receiving the response and (2) the real-world nature of the survey in asking opinions based on a specific inquiry/response to a specific company versus a general recollection over the previous 12 months.

In this study, 78–87% of HCPs who received information provided a quality rating of 4 or 5, on a 5-point Likert scale for trustworthiness, conciseness, completeness, clarity, relevance, and timeliness. Quality indicators did show some differences compared to the previous study by Albano et al. [7]. In both the Albano study and this current study, all quality indicators were rated as a 4 or 5 by majority of HCPs; however, in this current study the ratings were 2 to 20% higher than in Albano. Specifically, 62% of HCPs rated conciseness as a 4 or 5 in the previous study compared to 82% in the current study. Completeness increased from 72 to 77%; relevance from 77 to 79%; clarity from 73 to 80%; trustworthiness from 65 to 84%; and timeliness from 71 to 86%. In a post-hoc analysis all the quality indicators in the current survey were statistically significantly higher than in the Albano study, *p* < 0.001 via the Mann–Whitney test. These increases are most likely due to the real-time nature of the survey in relationship to the receipt of the response to the HCP’s question.

The reason for medical information inquires did not vary by company or by HCP type. The most common reasons for requesting information were to advance knowledge or education of the HCP (110/228, 48%), followed closely by the care of a current (60/228, 26%) or future patients (60/228, 26%). These settings were similar in order, but with a smaller total percentage to that seen in Albano [7]. The numerical differences seen may have been due to context of the two surveys, as Albano was relying on recall over the previous 12 months with potentially multiple contacts compared to a single, more recent inquiry in this survey.

When queried on the relevance of the information in their practice the most common answers were for their own education (99/226, 44%), or to share with peers (91/226, 40%), followed by treatment decisions (88/226, 39%) and patient discussions (64/226, 28%). There were statistically significant differences seen between nurse practitioners and pharmacists or physicians for several of the relevancies including leading to a prescribing decision and use in discussions with a patient. Additionally, pharmacists were more likely to share the information with a peer compared to physicians. Similar to the findings in the reason for the requests, in comparison with Albano [7], the specific numbers were lower in this survey with the same aforementioned rationale. By understanding these differences, Med Info Groups can better assist and tailor the information to the HCP types.

The impact on patient care was focused on enabling the HCP to educate patients more effectively (86/222, 39%), as well as treatment efficacy enhanced (70/222, 32%), and other positive impact (65/222, 29%). The avoidance of drug-drug interactions was rated lowest (18/222, 9%). Overall, these percentages are lower than seen in Albano [7]; however, in this survey HCPs were only responding to one request for information whereas in Albano they were considering requests made in the last 12 months. In addition, for the responses that the information did not enhance or impact patient care, again timing may have been an issue. The survey link was sent with the response so the full impact of the information may not have been seen by the time the survey was completed. There were statistically significant differences seen amongst HCPs for several of the impacts including enhancing treatment efficacy, avoided potentially harmful drug-drug interactions, educating patients more effectively, clarifying appropriate dosing regimen, and educating patients with comorbidities. Understanding these differences can aid the Medical Information Specialist in developing responses to various HCP types.

While nurse practitioners only accounted for 9% of the respondents, they noted significant differences in both the relevance and impact of the information especially in educating patients and prescribing decisions which includes clarifying dosing regimens.

### Limitations

While this survey provides an evaluation of Medical Information Services from the eight pharmaceutical companies, it is only a snapshot of the broader Medical Information Services community. Additionally, the sample size was limited to the responses received and varied based on the specific company. Overall, 40% were pharmacists, 28% physicians, and 18% were either nurse practitioners or registered nurses. There was 15% from a private practice setting and 41% from other. Given these numbers, there were limited data to allow for any in-depth analysis among all of the different characteristics. The survey was not tied to the specific question asked, so there was no analysis of the impact based on question type or specific product. This may be an area of further study. In addition, any of the “other” responses did not have an open text field for clarification due to concern of adverse events and required reporting.

## Conclusion

Healthcare professionals use medical information to assist them in completing their daily responsibilities of making treatment decisions and providing optimal patient care. The value of medical information from the pharmaceutical company is difficult to quantify. Understanding the quality assessment, utilization, and the impact on patient care by HCPs can provide a broad descriptor of value and identify opportunities for improvement of the medical information responses. This study supports the value, quality, and utility of the medical information responses provided by pharmaceutical companies to HCPs in their practice(s).

Pharmaceutical company Med Info Groups are continuously seeking opportunities to improve the medical information they provide to HCPs. Based on these findings, Med Info Groups can better tailor the responses they are providing to fit the needs of the HCP accessing the information. The actionable insights derived from this study can enhance the responses that Med Info Groups provide as there is a better understanding on how HCPs are using the information they receive from a range of pharmaceutical companies. Additionally, this information can be used internally to demonstrate the value of Medical Information and how providers are using the information to optimize patient care.

## Supplementary Information

Below is the link to the electronic supplementary material.

Supplementary file1 (DOCX 23 KB)
