# Supplementary material for: Enhancing Patient Care II: The Clinical Impact of Medical Information Services
Source: Ther Innov Regul Sci. 2022 Mar 15;56(3):483–91. doi: 10.1007/s43441-022-00385-1 (PMC8964574; doi:10.1007/s43441-022-00385-1)
Supplement: Supplementary file 1 — Supplementary file1 (DOCX 23 KB) [file 43441_2022_385_MOESM1_ESM.docx]

Appendix A: Survey questions

**PhactMI Clinical Impact Survey - COMPANY**

**Clinical Impact of Medical Information from COMPANY Medical Information**

You are being asked to participate in a quality improvement project evaluating the clinical impact of medical information obtained from COMPANY Medical Information. This is a voluntary survey and no personal identifiers will be collected. It is your choice to take part or not.

The purpose of this study is to collect survey data regarding healthcare providers’ (HCPs) opinions concerning the clinical impact of medical information provided by COMPANY Medical Information.

The online survey will take about 5 minutes to complete. 

Your participation is voluntary. If you choose to take part now, you may change your mind and withdraw later. If you do not click on the ‘submit’ button after completing the form, your responses will not be recorded. You may also choose to skip any questions that you do not wish to answer. However, once you click the ‘submit’ button at the end of the form, your responses cannot be withdrawn as we will not know which ones are yours.

#### **1. What type of healthcare provider are you?**

- MD/DO
- RPh/PharmD
- NP/APN
- RN
- PA
- Other

#### **2. What is your specialty?**

- Primary Care
- Cardiology
- Endocrinology
- Pulmonology
- Oncology/Hematology
- Orthopedics
- Neurology
- Rheumatology
- Psychiatry
- General Surgery
- Other

#### **3. In which of the following settings do you practice? (Select all that apply.)**

- Academic/teaching hospital
- Community hospital
- Outpatient Clinical Practice (e.g. HMO)
- Private Practice
- Pharmacy – Hospital
- Pharmacy – Retail
- Managed Care
- Research
- Long-term care
- I’m not currently practicing
- Other

#### **4. How many years have you been practicing?**

- 1-2 years
- 3-5 years
- 6-10 years
- 11-20 years
- ˃ 20 years

#### **5. How many times have you used COMPANY Medical Information Services in the past 6 months?**

- 1-5 times
- 6-10 times
- 11-15 times
- >15 times

#### **6. Overall, how would you rate the quality of information you have received from COMPANY Medical Information services (Rate each on a scale of 1 to 5, 5 being highest)?**

|  | 1 (lowest) | 2 | 3 | 4 | 5 (highest |
| --- | --- | --- | --- | --- | --- |
| Timeliness of response |  |  |  |  |  |
| Relevance of response |  |  |  |  |  |
| Clarity of response |  |  |  |  |  |
| Completeness of response |  |  |  |  |  |
| Conciseness of response |  |  |  |  |  |
| Trustworthiness of response |  |  |  |  |  |

#### **7. In what settings have you contacted COMPANY to obtain medical information? (Select all that apply)**

- During an emergency situation
- At the point of care for a specific patient
- For a specific patient which I have in mind (not at point of care)
- To reflect on a treatment decision where I used a particular drug
- To advance my knowledge or education
- To gain understanding of the cost of treatment
- Other

#### **8. Regarding the pharmaceutical medical information you have received from COMPANY, how was this relevant to your practice? (Select all that apply)**

- Led to treatment management decision (other than prescribing)
- Led to prescribing decision
- Used in discussions with patients
- Used for future treatment decisions
- Used to determine patient’s insurance coverage and/or out-of-pocket costs
- Used to enroll patients in clinical trials
- Used information for own education
- Shared information with peers
- Other

#### **9. In your clinical opinion, how has receiving medical information from COMPANY enhanced patient care in your practice? (Select all that apply)**

- Efficacy of treatment regimen was enhanced
- Avoided adverse events
- Avoided potentially harmful drug-drug interactions
- Able to educate patient more effectively
- Appropriate dosing regimen was clarified
- Educated about drug use in patient with comorbidities
- Facilitated patient access to treatment
- Enhanced patient adherence
- Other positive impact
- Patient care was not enhanced by information
